# High Resolution MEMS Accelerometers to Estimate VO_2_ and Compare Running Mechanics between Highly Trained Inter-Collegiate and Untrained Runners

**DOI:** 10.1371/journal.pone.0007355

**Published:** 2009-10-06

**Authors:** Stephen J. McGregor, Michael A. Busa, James A. Yaggie, Erik M. Bollt

**Affiliations:** 1 Applied Physiology Laboratory, Eastern Michigan University, Ypsilanti, Michigan, United States of America; 2 College of Health Professions, University of Findlay, Findlay, Ohio, United States of America; 3 Department of Mathematics & Computer Science, Clarkson University, Potsdam, New York, United States of America; Karolinska Institutet, Sweden

## Abstract

**Background:**

The purposes of this study were to determine the validity and reliability of high resolution accelerometers (HRA) relative to VO_2_ and speed, and compare putative differences in HRA signal between trained (T) and untrained (UT) runners during treadmill locomotion.

**Methodology:**

Runners performed 2 incremental VO_2max_ trials while wearing HRA. RMS of high frequency signal from three axes (VT, ML, AP) and the Euclidean resultant (RES) were compared to VO_2_ to determine validity and reliability. Additionally, axial rms relative to speed, and ratio of axial accelerations to RES were compared between T and UT to determine if differences in running mechanics could be identified between the two groups.

**Principal Findings:**

Regression of RES was strongly related to VO_2_, but T was different than UT (r = 0.96 vs 0.92; p<.001) for walking and running. During walking, only the ratio of ML and AP to RES were different between groups. For running, nearly all acceleration parameters were lower for T than UT, the exception being ratio of VT to RES, which was higher in T than UT. All of these differences during running were despite higher VO_2_, O_2_ cost, and lower RER in T vs UT, which resulted in no significant difference in energy expenditure between groups.

**Conclusions/Signficance:**

These results indicate that HRA can accurately and reliably estimate VO_2_ during treadmill locomotion, but differences exist between T and UT that should be considered when estimating energy expenditure. Differences in running mechanics between T and UT were identified, yet the importance of these differences remains to be determined.

## Introduction

There has been increasing interest in recent years in the use of technology to assess training load in runners in the field. Efforts have been made to utilize global positioning system (GPS) devices to record running speed and estimate expenditure relative to speed [1], but these devices exhibit relatively low resolution, and some problems inherent to the technology have yet to be resolved (e.g. altitude errors, errors on curved courses) [2], [3]. In particular, characteristic errors on curved courses is problematic as many running events and training sessions are performed on tracks, and devices used to assess workload need to be accurate and precise under these conditions. A possible candidate that might serve as a means to assess training load for running is the accelerometer. The use of accelerometers to measure human movement has increased greatly in recent years [4], [5], [6], but, from a physiological perspective, they have commonly been used as “activity monitors” for the coarse-grained measurement of gross movements. In many cases, the goal of using these low resolution accelerometers has been in attempt to objectively determine energy expenditure during free living non-formal activities [7]. One problem with these devices is a lack of standardization as output is often reported as activity counts, which are determined by subjective criteria applied during the data conditioning process. The lack of standardization has led to numerous studies that have been performed in attempts to develop regression equations to fit activity counts obtained from these devices to other measures of metabolic work (e.g. VO_2_ and/or doubly labeled water) [6], [8], [9]. This is problematic when attempting to compare results between studies using different accelerometers, or using the same accelerometers, but different criteria for activity counts. In this regard, Corder et al. [10] has recommended greater transparency and standardization in the reporting of accelerometry data. In particular, Corder et al. has recommended using acceleration reported in standard units such as m/s^2^ or g's as opposed to activity counts or other conditioned data outputs based on arbitrary threshold criteria, which are often proprietary and manufacturer specific. This would facilitate greater scientific transparency and cross-study comparisons.

Although clinical/epidemiological studies using accelerometers are numerous, there have been few attempts to use this approach in athletic populations in order to objectively quantify external work of a dynamic activity such as running. Fudge et al. [11] did investigate the relationship between activity monitor accelerometers and VO_2_ in trained runners, but determined that a correction for HR was necessary to obtain strong correlations. Further, some of the accelerometers tested would not show a strong correlation with VO_2_ during running even with a correction for HR [11]. So, although the utility of accelerometer based activity monitors for the measurement of “work” in running has been investigated with some success, it might be expected that sophistication could be improved relative to this through the use of devices exhibiting higher resolution. Further, in an activity such as running, portable accelerometers might not only serve as ergometers to measure work, but some insight might be gained by using the high frequency signal from such a device to examine running mechanics collected during “real world” activities such as racing and training.

Accelerometers have been used in the field of biomechanics for decades [12], [13] for the purposes of gait analysis. In contrast to “activity monitors” used for metabolic/activity studies, these devices generally collect data at higher frequencies in continuous, as opposed to discretized fashion and as such, provide higher resolution. The high resolution accelerometers (HRA) provide some advantages over traditional approaches (e.g. force plate analysis or inverse dynamics). In particular, HRAs are portable, light, and generally can be used to either stream data at high frequency in real time, or datalog similarly high frequency signals collected during “real world” activities of locomotion that would not be possible using other means. HRA used for gait analysis have previously been limited by data storage capacity and portability, with the recent innovation of microelectromechanical system (MEMS) accelerometers, the aforementioned advantages may be exploited to a greater extent. In particular, it would be of interest to identify putative differences in the characteristics of “good” runners versus comparatively poorer runners using HRA devices. Others have examined differences in mechanics of running between trained and untrained individuals, but little definitive information has been obtained [14], [15], [16], [17], so, HRA might provide a unique perspective in this regard.

Therefore, the purposes of this study were to use highly trained collegiate runners and untrained individuals to determine 1) the validity of HRA reported in g's to VO_2_ and speed, 2) the test-retest reliability of HRA across a wide range of walking/running speeds, and 3) differences in HRA between trained and untrained runners during treadmill locomotion. It was hypothesized that the sensitivity provided by HRA would allow identification of differences between these two groups that might provide insight into differences in running mechanics between trained and untrained runners.

## Methods

### Subjects

Eighteen subjects consisting of nine male NCAA Intercollegiate Division 1 distance runners (T) and nine recreationally active, college students considered untrained (UT) for running (Table 1) gave written informed consent to take part in this study, which was approved by the Eastern Michigan University College of Health and Human Services - Human Subjects Review Committee. Criteria to be considered UT was running less than four times per week and an estimated 10 km performance time of greater than 45 min.

**Table 1:** Physical characteristics of the subjects.

	Mass (kg)	Height (cm)	Age (yr)	VO2max (ml/kg/min)
T	65.5 (5.7)	181.8 (4.1)	21.4 (1.7)	70.1 (6.2)
UT	69.8 (11.8)	176.9 (5.7)	31.6 (9.6)	49.2 (5.0)

T – Trained collegiate runners (n = 7), UT – untrained runners (n = 7). Values are mean ± (SD).

### Experimental Design

Subjects completed two continuous, incremental exercise tests on a motorized treadmill (True ZX-9, St. Louis, MO) with at least 6 days separating each trial. Exercise tests were performed to volitional exhaustion while high resolution triaxial acceleromety (HRA) and metabolic gasses were collected to determine relationships between, HRA, VO_2_, walking and running speed. In addition, validity and reliability of the unfiltered, HRA was determined. After the first trial, two T subjects could not complete a second trial due to injury. Data for these subjects was therefore not included in the reliability analysis, but was used for correlations and regression curve fits. Similarly, two UT subjects could not complete the 16 km/h stage and, therefore, their data was also excluded to enable balanced comparisons between T and UT for running stages. For all between group comparisons, only running speeds up to 16 km/h were used since this was a speed both UT (n = 7) and T (n = 7) could complete.

### Procedure

Subjects reported to the laboratory on the day of examination after a 3 hr fast and having refrained from strenuous exercise, alcohol, and caffeine for 24 hours prior to the day of testing. Height and body mass were measure upon arrival at the laboratory (Mettler-Toledo, OH).

### Incremental exercise test to volitional exhaustion

In each of the two tests, subjects began walking at 2 km/h and speed was increased 2 km/h every two minutes until volitional exhaustion. The treadmill grade was held constant at 1% to simulate normal over-ground walking/running. During tests, metabolic data was collected on a breath-by-breath basis using portable open circuit spirometry (Jaeger Oxycon Mobile, Viasys, CA). VO_2max_ was determined as the highest 30 s average of the test.

### Metabolic Measurements

Indirect calorimetry was used to collect breath-by-breath measurements of VO_2_ and VCO_2_ using electrochemical oxygen measuring cell (SBx) in an Oxycon Mobile and averaged over 5 sec. Heart rate was collected continuously via telemetry using a Polar coded transmitter belt (Polar t-31, Polar Electro, Oulu, Finland). The oxygen and carbon dioxide sensors were calibrated prior to each test for: ambient conditions (temperature and barometric pressure), volume and gas content against precision analyzed gas mixtures (16% O_2_ and 4% CO_2_). The oxygen cost of locomotion (O_2_C) was determined by the VO_2_ at the given speed corrected for resting VO_2_ (ml/kg/min) expressed relative to speed (km/h).

### Accelerometry

The HRA device, a triaxial microelectromechanical MEMS accelerometer model ADXL210 (G-link Wireless Accelerometer Node ± 10 g Microstrain, Inc., Williston, VT) was placed anatomically at the intersection of the sagittal and axial planes on the posterior side of the body in line with the top of the iliac crest in order to approximate the center of mass [18]. The accelerometer was mounted to a semi-rigid strap and additionally secured with elastic tape in order to any extraneous movement not associated with locomotion. Acceleration in g's was streamed in real time using telemetry to a base station at a frequency of 625 Hz.

### Data Analysis

Raw accelerometry signal (g) was saved in Agilelink software (Microstrain, VT) and exported to Signal Express software (Labview, TX) in ASCII format. Full length files were parsed into 1 min segments and dynamic accelerations correcting for dynamic accelerations were extracted according to Halsey [19]. The last one minute of each treadmill stage was used to calculate Root Mean Square (RMS) value using Signal Express for each axis, vertical (VT), lateral (ML), anterior/posterior (AP), and Resultant (RES). The RES value was calculated according to the equation
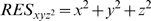
(1)Where x, y and z equal the Vertical, Lateral and Anterior/Posterior axes, respectively.

The 1 minute RMS of acceleration were generated using Signal Express and compared to the 1 minute average of VO_2_ for the last minute of each corresponding stage. Comparisons were made using Pearson's product correlation, RMS of raw signal were also compared to VO_2_ using a linear regression curve fit. Validity and reliability of the HRA were determined by calculation of coefficient of variation (CV), test-retest reliability (R) and Interclass Correlations (ICC) (SPSS, IL; α = 0.05).
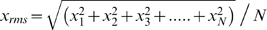
(2)


### Economy of Accelerations

For each axis (VT, ML, AP and RES), an economy value was established by the calculation
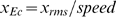
(3)where X is the respective axis (e.g. VT, ML, AP, or RES) and speed is the speed of the stage being calculated. This resulted in economy of acceleration relative to speed for each axis in the vertical (VT_Ec_), mediolateral (ML_Ec_), anterior posterior (AP_Ec_) and resultant (RES_Ec_) in g/km/h.

### Ratio of Accelerations Relative to RES

To determine the contribution of accelerations specific to each axis as a proportion of RES accelerations, values were calculated by 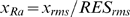
(4)Where X is the respective axis (e.g. VT, ML, and AP). This calculation resulted in a unitless ratio for each axis, VT_Ra_, ML_Ra_ and AP_Ra_.

## Results

### Regressions

**Figure 1:**
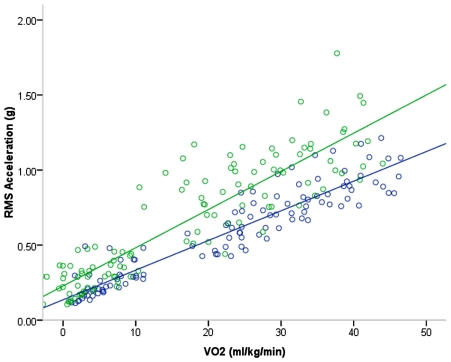
Regressions of VO_2_ versus RES_rms_ acceleration during walking and running in highly trained (T) and untrained (UT) runners. Green points – UT individual values. Green line – UT regression (r = 0.92; *p*<.001). Blue points – T individual values. Blue line – T regression (r = 0.96; p<.001).

Results of regression curve fits of accelerometry vs. VO_2_ can be seen in Table 2 and Figure 1. Linear, quadratic and cubic regressions were attempted for VO_2_ against each axis, and in all cases, quadratic and cubic regressions were not more significant than linear. It is readily apparent that the prediction of VO_2_ when regressed to the VT axis exhibited the weakest relationship across the entire range of speeds tested. When VO_2_ was regressed against A/P and RES, similarly strong R values were observed, although the RES was much more significant as evidenced by the F values (Table 2). When UT and T groups were regressed for RES against VO_2_ though, it can be seen in Figure 1 that the relationship was stronger in T than in UT (*R* ^2^ = 0.9 vs 0.85, respectively).

**Table 2:** Regression parameters of VO_2_ vs individual axes.

Axis	Equation	F	*R*	Adjusted *R^2^*
VT	Linear	444.7	.87	.751
	Quadratic	221.0	.87	.750
	Cubic	221.2	.87	.750
ML	Linear	496.3	.88	.771
	Quadratic	420.3	.92	.851
	Cubic	280.1	.92	.851
AP	Linear	1242.3	.95	.894
	Quadratic	668.3	.95	.900
	Cubic	445.1	.95	.900
RES	Linear	1213.5	.95	.892
	Quadratic	602.9	.95	.891
	Cubic	603.4	.95	.891

### Reliability

The reliability of the HRA and VO_2_ instruments used for this study are presented in Table 3. It can be seen that the test-retest reliability was quite high for all axes, but highest for RES, which was comparable to VO_2_. Further evidence of the reliability of using RES is demonstrated by the CV which was also on par with VO_2_. On the other hand, CVs were quite high in the ML and AP axes.

**Table 3:** Reliability parameters for test-retest conditions and VO_2_.

Axis	ICC	CV	Pearson's R
VT	.98	5.7	.96
ML	.97	23.7	.95
AP	.97	23.7	.94
RES	.99	5.1	.98
VO_2_	.99	5.2	.98

### Comparison of Trained (T) vs Untrained (UT) walking and running mechanics by HRA

In order to determine if HRA may be useful to provide information aside from estimation of VO_2_, such as differences in running mechanics between groups based on training status, the data was compared between trained (T) and untrained (UT) runners. Comparisons included 1) RMS of acceleration, 2) Economy of acceleration and 3) Ratio of acceleration of individual axes with RES. Initial analyses showed a speed by training interaction for several of the parameters as a function of speed across the entire speed spectrum. Further, some parameters (e.g. VT_Ec_ and VT_Ra_) appeared to exhibit dramatic changes from walking to running, therefore, data were analyzed for walking and running phases separately.

### WALK ONLY COMPARISONS

#### RMS of Accelerations

To clarify the differences between groups with regard to accelerations during walking, RMS of accelerations were compared between T and UT (Table 4) for walk stages only (2–6 km/h). Main effects were observed for speed across all axes (VT, ML, AP and RES; p<.001), but no effects for training, or speed by training interactions were observed.

**Table 4:** Acceleration parameters versus speed in trained (T) and untrained (UT) runners during walk stages only.

			2	kph	4	kph	6	kph	
RMS	VT	T	0.02	(.01)	0.02	(.01)	0.07	(.01)	*
		UT	0.02	(.01)	0.01	(.01)	0.07	(.01)	*
	ML	T	0.10	(.01)	0.13	(.01)	0.19	(.01)	*
****		UT	0.08	(.01)	0.12	(.01)	0.19	(.01)	*
	AP	T	0.16	(.03)	0.20	(.02)	0.24	(.02)	*
		UT	0.17	(.03)	0.21	(.02)	0.28	(.02)	*
	RES	T	0.19	(.03)	0.24	(.02)	0.32	(.02)	*
		UT	0.20	(.03)	0.24	(.02)	0.36	(.02)	*
Economy	VTEc	T	0.01	(.00)	0.00	(.00)	0.01	(.00)	*
		UT	0.01	(.00)	0.00	(.00)	0.01	(.00)	*
	MLEc	T	0.05	(.03)	0.03	(.00)	0.03	(.00)	*
****		UT	0.04	(.03)	0.03	(.00)	0.03	(.00)	*
	APEc	T	0.08	(.01)	0.05	(.01)	0.04	(.01)	*
		UT	0.09	(.01)	0.05	(.01)	0.05	(.01)	*
	RESEc	T	0.09	(.01)	0.06	(.01)	0.05	(.01)	*
		UT	0.10	(.01)	0.06	(.01)	0.06	(.01)	*
Ratio	VTRa	T	0.07	(.02)	0.07	(.02)	0.19	(.02)	*
		UT	0.10	(.02)	0.07	(.02)	0.18	(.02)	*
	MLRa	T	0.57	(.03)	0.56	(.03)	0.60	(.03)	†
****		UT	0.49	(.03)	0.50	(.03)	0.56	(.03)	†
	APRa	T	0.80	(.02)	0.81	(.02)	0.76	(.02)	†
		UT	0.84	(.02)	0.86	(.02)	0.80	(.02)	†

Values are mean ± (SE) * - significant effect for speed (p<.05), † - significant effect for training (p<.001).

#### Economy of Accelerations

Economy of accelerations were examined between T and UT (Table 4) during the walk stages only (2–6 km/h), and significant main effects were observed for speed (VT, ML, AP and RES; p<.001) but not for training. No speed by training interactions were present for economy of accelerations during the walking stages.

#### Ratio of Accelerations

Accelerations during the walk only phase (2–6 km/h) were expressed as a ratio of axial accelerations relative to RES and compared between T and UT (Table 4). Significant main effects were present for speed for VT (p<.001), but not ML (p = .03) and AP (p = .07). Also, a training effect was present for ML and AP (Table 4). There was no speed by training interaction for ratio of accelerations during the walking stages.

#### Metabolic parameters

Although there were few main effects for training between groups, and no speed by training interaction for any of the acceleration parameters examined during the walking stages, there were significant main effects for speed and training observed for VO2 and O2C (p<.001), as well as a speed by training interaction (p = .003). Both VO2 and O2C were higher for T vs UT, meaning oxygen consumption was higher at given walking speed in T vs UT (Table 5). Because a training effect was also present for the respiratory exchange ratio (RER), energy expenditure (kcal/kg/min) was calculated based on the relationship between RER and VO2, and a training effect was still present. This indicated T expended more energy while walking compared to UT.

**Table 5:** Metabolic parameters in trained (T) and untrained (UT) runners during walk stage only.

		2	kph	4	kph	6	kph	
VO2	T	2.75	(0.38)	5.14	(0.36)	9.38	(0.38)	*†
(ml/kg/min)	UT	0.57	(0.38)	3.06	(0.38)	7.84	(0.38)	*†
VO2	T	179.61	(29.8)	337.02	(28.72)	614.54	(29.8)	*†
(ml/min)	UT	47.90	(29.8)	231.31	(28.72)	596.07	(29.8)	*†
RER	T	0.79	(0.02)	0.77	(0.02)	0.76	(0.02)	†
	UT	0.83	(0.02)	0.83	(0.02)	0.81	(0.02)	†
EE	T	1.4E-02	(1.9E-03)	2.6E-02	(1.8E-03)	4.8E-02	(1.9E-.03)	*†
(kcal/kg/min)	UT	2.9E-03	(1.9E-03)	1.6E-02	(1.8E-03)	4.0E-02	(1.9E-.03)	*†

*- significant effect for speed (p<.05), † - significant effect for training (p<.05).

### RUN ONLY COMPARISONS

#### RMS of Accelerations

**Figure 2:**
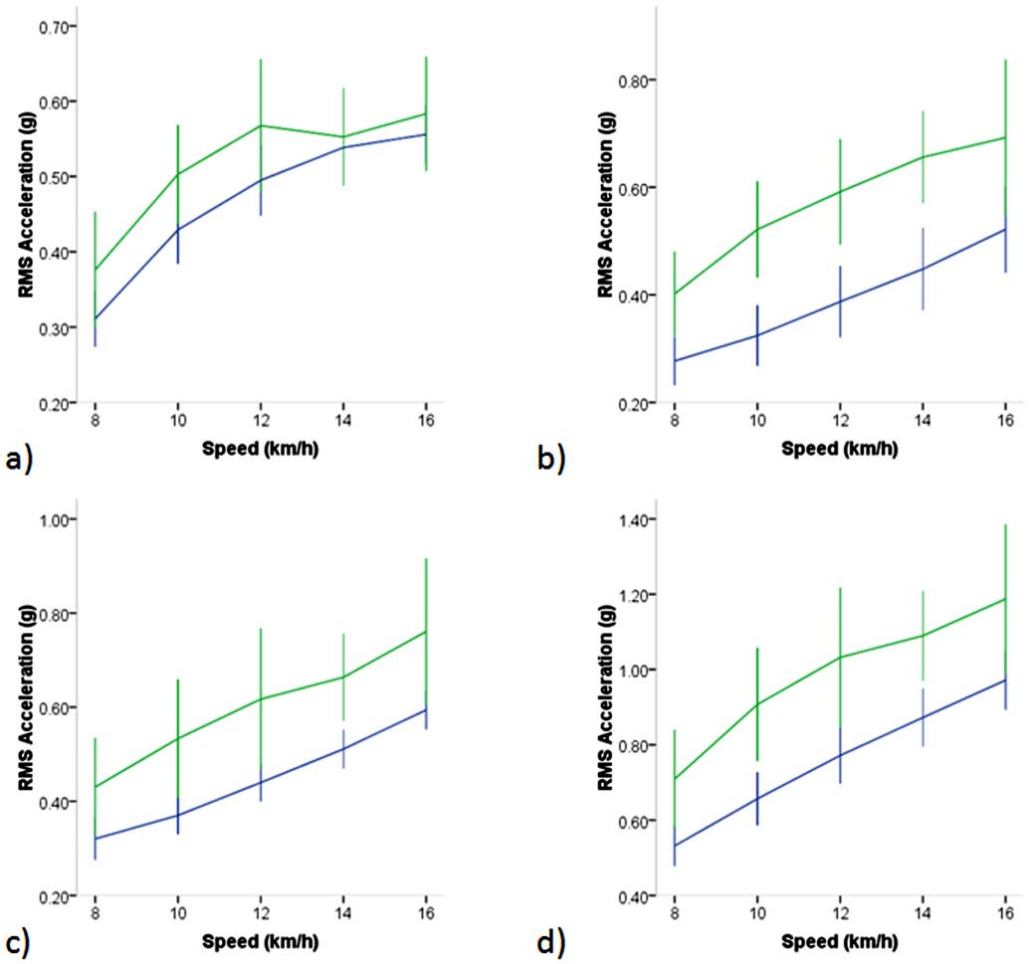
RMS of accelerations (g) for individual axes versus speed in highly trained collegiate (Blue) and untrained (Green) runners during the run stages only. a) VT b) ML c) AP d) RES. Significant effects for speed (p<.001), and for training present in all axes (p<.001).

To clarify the differences between groups with regard to accelerations during running, RMS of accelerations were compared between T and UT (Figure 2) for run stages only (8–16 km/h) in similar fashion to the walk only stages. In this case, main effects were observed for speed across all axes (VT, ML, AP and RES; p<.001). In contrast to walk only stages though, main effects for training were observed for all axes (VT, ML, AP and RES; p<.001), and RMS of accelerations for T were lower than UT in each case. No speed by training interactions were present.

#### Economy of Accelerations

**Figure 3:**
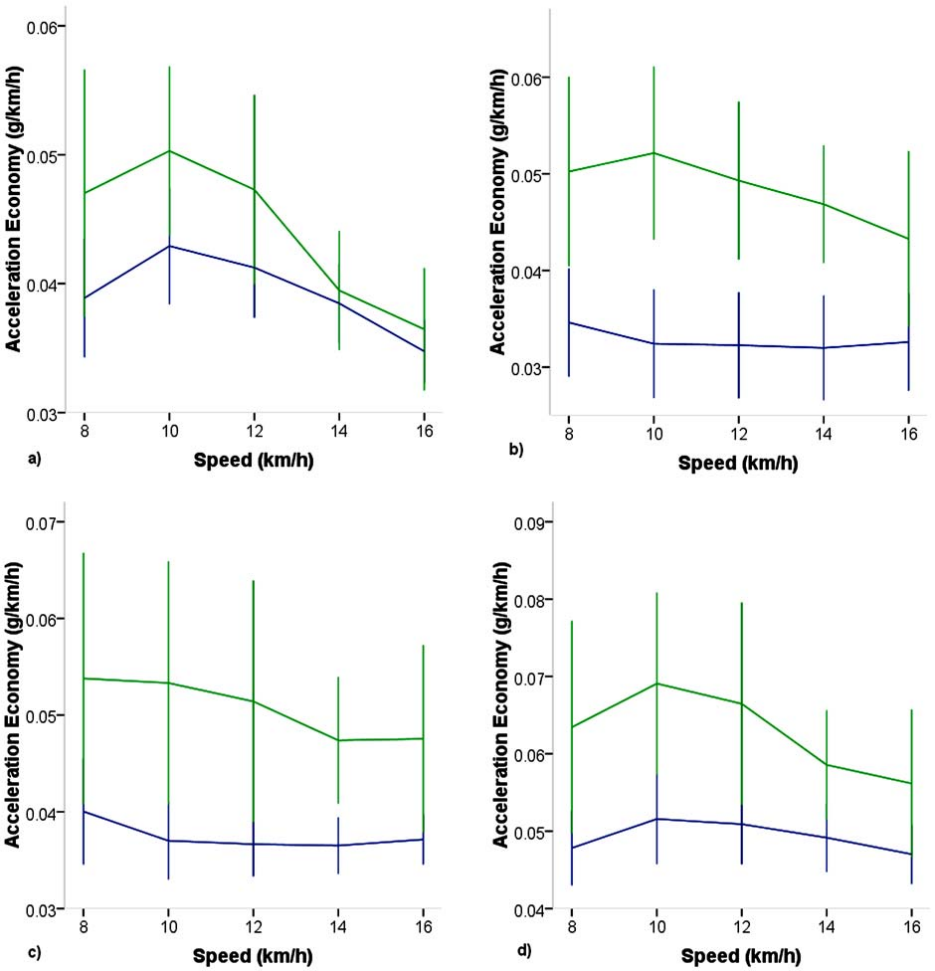
Economy of acceleration for individual axes versus speed in highly trained collegiate (Blue) and untrained (Green) runners during the run stages only. a) VT b) ML c) AP d) RES. Significant effects for speed in VT and RES (p<.001) and for training present in all axes (p<.001).

When accelerations were expressed as economy relative to speed for the run only stages (8–16 km/h) and compared between T and UT (Figure 3), significant main effects were observed for speed in the VT and RES (p<.001), but not ML and AP axes (p>.62). Significant effects for training were observed for VT (p = .002), ML, AP, and RES (p<.001), but no speed by training interaction was observed in any axis. When a training effect was present for economy of acceleration it was lower in T than in UT for each case.

#### Ratio of Accelerations

**Figure 4:**
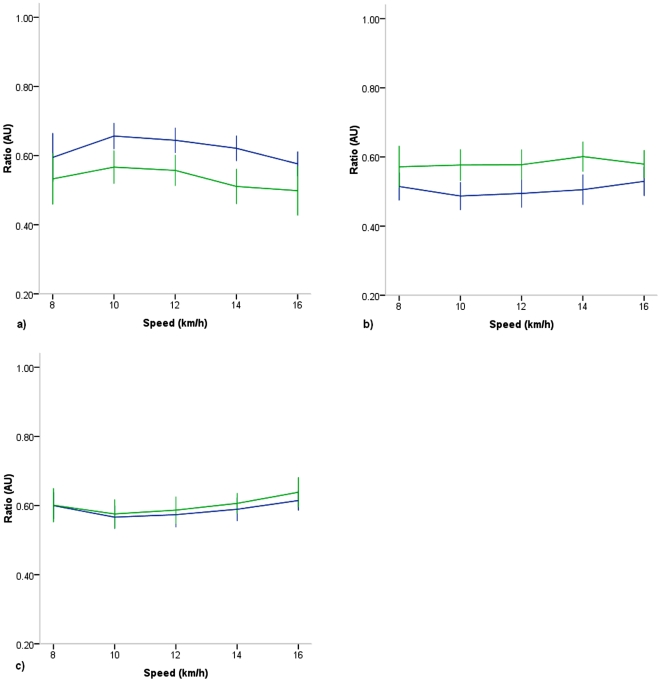
Ratio of acceleration for individual axes versus speed in highly trained collegiate (Blue) and untrained (Green) runners during the run stages only. a) VT b) ML c) AP. Significant effects for speed (p<.001) and for training present in all axes (p<.001).

When accelerations were expressed as a ratio of axial accelerations relative to RES (Figure 4), significant main effects were present for speed in all axes (p<.001) as well as for training VT and ML (p<.001), but not AP (p = .243). Interestingly, in contrast to other parameters where each variable was lower in T than in UT, in this case, VT was higher in T than in UT, while ML was lower in T than in UT. No speed by training interaction was observed.

#### Metabolic parameters

**Figure 5:**
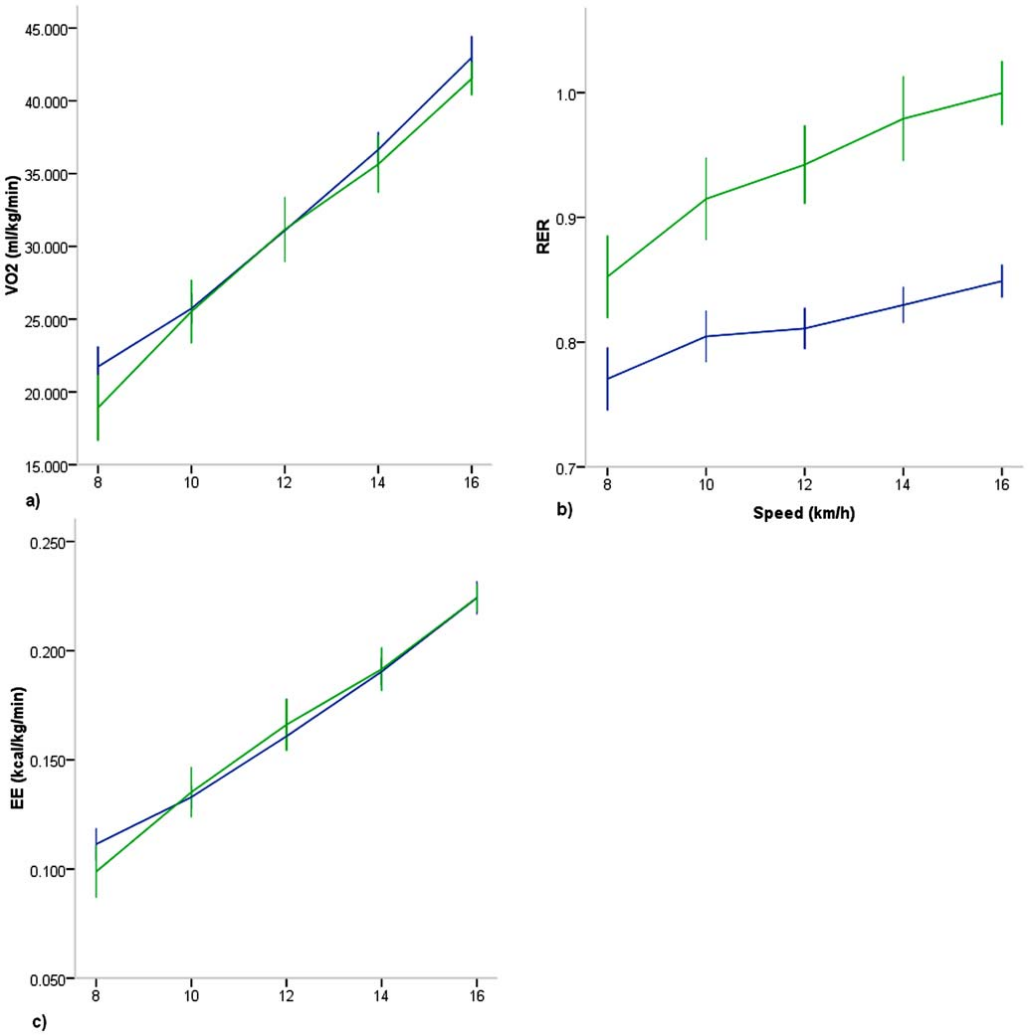
Metabolic parameters in highly trained collegiate (Blue) and untrained (Green) runners during the run stages only. a) VO2 b) RER c) Energy Expenditure. Significant effects for speed for all parameters (p<.001). Significant effects for training for RER (p<.001) and VO_2_ (p = .03). Training effect for Energy Expenditure not significant (p = .76).

When VO2 and O2C were expressed relative to speed for run only stages between T and UT (Figure 5), significant main effects were observed for speed (p<.001) and training (VO2, p = .03; O2C, p = .004). No speed by training interaction was present. Similarly to the walking stages, these effects were due to a higher VO2 and O2C in the T vs UT groups, meaning, T runners consumed more oxygen at given speed compared to UT. As with the walk only stages, there was a training effect in RER between T and UT (p<.001), but in contrast to the run only stages, when energy expenditure (kcal) was compared between groups, no training effect was present (p = .902; Figure 5). Thus, although the T consumed more oxygen at a given running speed, more fat was oxidized relative to UT, and hence, energy expenditure was not different between groups.

## Discussion

Here we report the relationship of HRA to VO_2_ across a range of walking and running speeds in highly trained runners compared to untrained runners. Further, this is the first study to demonstrate distinct differences in accelerations in different axes between trained and untrained individuals while running and walking that are not reflective of differences in energy expenditure.

### Validity and Reliability of the HRA

It was determined that the rms of RES acceleration value calculated from the three individual axes exhibited a strong relationship with VO_2_ (Figure 1), strong internal validity and strong test-retest reliability (Table 3). These data indicate that HRA may prove of value for monitoring training load in trained runners in similar fashion to portable HR monitors, while providing additional information on gait characteristics, and changes in speed with good accuracy and reproducibility. It should be noted though, that there were significant differences in acceleration parameters between trained and untrained groups that were not reflective of differences in VO_2_ and energy expenditure. Therefore, caution should be taken when using HRA in heterogeneously trained populations to estimate VO_2_ and/or energy expenditure. In homogenous populations of trained runners though, these devices seem appropriate for this purpose.

### Comparison of Trained (T) vs Untrained (UT) walking and running mechanics by HRA

#### Walking

It was interesting to note that, during walking, there were no significant differences between groups in RMS of acceleration or economy of acceleration, nor was there an interaction between training and speed for these parameters; despite the fact that there were significant differences in VO2 and O2 cost (O2C) between groups. There was a significant group effect for ratio of accelerations relative to RES (ML, AP, but not VT) between T and UT due to training status, although the significance of this observation is unclear. Moreover, it was unexpected to observe that the O2C was actually higher in T vs UT. Substrate utilization (e.g. carbohydrate vs lipid) can impact VO2 and there was a significant training effect for RER as well as VO2, therefore, when energy expenditure was determined accounting for VO2 and RER, there was still a significant training effect between groups. This indicated that cost of walking was higher for T compared to UT both in terms of VO2 and energy expenditure. Therefore, although HRA are valid and reliable instruments for the assessment of work relative to speed, caution should be exercised when using these devices to compare energy expenditure between trained and untrained individuals during walking.

#### Running

During running, T runners exhibited lower RMS of acceleration values at all speeds relative to UT runners (Figure 2). This is consistent with the notion that good runners exhibit better “economy” relative to poorer runners. By minimizing accelerations at any given speed, good runners would presumably use less energy to maintain a given constant speed, an observation reported by some investigators [15], [20], [21]. This is a controversial view though, and in the current study, differences in RMS of accelerations did not result in reduced O2C or energy expenditure in T versus UT, which is agreement with some previous reports [16], [22]. It has been argued that reported differences in O2C between runners can be attributed solely to anatomical differences, as opposed to mechanical differences in running technique [23]. We did not investigate anatomical differences in this study, but it was of interest to further elucidate the differences in running mechanics between these groups.

To investigate this relationship further, when economy of accelerations were examined, an inverse relationship to running speed for VT and RES was observed, while economy of ML and AP exhibited no relationship to speed (Figure 3). Further, economy measures (i.e. accelerations relative to speed) for each of these parameters were significantly lower in T than in UT runners. In addition to economy of acceleration, ratio of axial acceleration relative to RES was examined and significant effects were observed for speed and training (Figure 4). Although RMS and economy of accelerations in the VT axis were both lower in the T vs UT runners, the training effect in the VT axis was unique in that the T showed greater ratio of VT to RES when compared to UT. On the other hand, the ratio of ML and AP to RES were lower in T compared to UT (Figure 4). So, although T accelerated less than UT in the VT axis, a greater proportion of the overall accelerations were distributed to the VT axis. This phenomenon has not previously been reported and it is unclear what the putative value of such a difference would be in trained runners. Others have reported lower potential energy [16] in trained than lesser trained runners, which would be consistent with lower accelerations in the VT axis in T group in the current study. Williams and Cavanagh reported non-significantly lower peak forces in the vertical axis in runners who consumed less O2 at a given running pace [17]. Again, in the current study though, the T runners, who exhibited lower rms and economy in the VT did not consume less O2 at any comparable speed than the UT.

Several studies have examined the economy of running relative to VO2. In general, these studies have reported a reduced O2 cost during running (increased economy) in trained vs untrained [15], or no difference [16], [22]. In the present study, although numerous acceleration parameters were lower in the T vs UT runners, this did not translate to decreased O2 cost of running. Further, when accounting for differences in substrate utilization, the energetic cost of running was not different between groups. This is in agreement with data from Slawinski who used kinematic approaches to examine the cost of running between T and lesser trained runners and saw no difference in total cost [16].

Since the differences in acceleration parameters between groups did not result in improved energetic cost or O2 cost of running in T vs UT, the value in these supposed adaptations is not clear. Reduced absolute acceleration in VT and AP would presumably reduce the impact forces on the runner's anatomical components during deceleration phases and this might reduce muscle injury and improve ability to recover between training sessions. It might also result in a decreased likelihood of incapacitating injury as a result of impact forces. The risk factors of running related injury are not well understood [24], but it is likely that reducing the impact forces experienced by reducing accelerations in the VT and AP axes in highly trained runners would afford some protection.

Previous work by Fudge et al. [11] examining the utility of accelerometers for the assessment of running workload relative to VO2 in trained runners at high running speeds (8–18 km/h) showed some promise. They reported reasonably strong predictions of VO2 in walking and running with triaxial activity monitor accelerometers, but these strong relationships required correction using HR. In the current study, no correction for HR was examined, and yet, stronger relationships were observed than in the Fudge et al. study when VO2 was regressed to RMS of accelerometer signal, in particular, when regressed to RES (Figure 1). In the case of activity monitor accelerometers such as those used for the Fudge et al. study, considerations such as the thresholds for determining activity counts, as well as filtering bands applied are important [4], [6], [25]. Recently, Halsey et al. [19] used a similar approach to the current investigation by mounting HRA on the lower back (as well as other sites) of humans and reported strong relationships with VO2 (l/min) during walking and running. In contrast to the current study, they used absolute VO2 (l/min) as the criterion measure, and as such, relationships were improved by adding subject weight as a covariate in regression analysis. In the current investigation, the use of relative VO2 (ml/kg/min) as the criterion inherently corrects for bodyweight, and therefore strong relationships were observed and VO2 was strongly predicted by acceleration when regressions were performed using only relative VO2 and RES. Neither of the aforementioned studies investigated reliability of the devices used, and the reproducibility of their measures within subjects. Importantly, we show in the present investigation that HRA is not only valid relative to VO2, but also reliable on re-test. This is in comparison with a report from Henriksen et al. [26] in which HRA mounted to the lower back and RMS of vector sum derived values exhibited ICCs of 0.81–0.85. In the current study, the ICCs for the analogous RES were higher (0.99; Table 4).

Potential applications of this work are significant on several levels. First, it is attractive to consider the use of HRA in the same sense as a traditional, downloadable HR monitors for the quantification of global training load as HRA would not be susceptible to some of the limitations to HR (e.g. dehydration, psychological motivation etc) [27]. This could be of particular value for the application of performance modeling approaches such as the training impulse (TRIMPS), with the use of a work output based metric as opposed to HR. The TRIMPS system has been used extensively in various sports [28], [29], [30], [31], including running [30] and various inputs other than HR. Recently, we used an impulse-response model of performance in an elite middle distance runner and observed strong relationships between model parameters and competition performances using speed determined from training logs as the model input [32]. Since this admittedly rudimentary approach was successful, it should be expected that using HRA would be at least as successful, if not more so. The current work with HRA is limited in its generalizability to over ground running as it was performed only at a 1% grade. Halsey et al. did examine HRA and gradient walking and found significant relationship to VO2, albeit weaker than on level ground [19]. Further, the differences in acceleration parameters observed in the current study between T and UT subjects indicate there may be changes in running mechanics over time that may influence the relationship between HRA and VO2, and this should be taken into consideration when using HRA to monitor training load over time.

Second, the same aforementioned differences in acceleration parameters between groups in this work indicate that mechanics of running may change in the long term with training, and HRA may prove a valuable tool to track and quantify these putative changes. Further longitudinal studies will be necessary to determine if this is indeed the case, but this would be a valuable use for HRA in this respect.

Finally, a potential implementation of these devices is for the application of complex frequency or non-linear dynamical analysis of such data to gain further insight into the nature of fatigue or the constraints of running. There has been some interest in the field of biomechanics with regard to high level mathematical (e.g. non-linear dynamical analysis, spectral analysis etc.) of walking/running gait patterns [33], [34], [35], [36], [37], [38], [39]. There have been a few attempts to extend some of these techniques to theories of fatigue in competitive running and this area potentially holds promise [40], [41], [42]. The use of HRA signal for these types of analysis may provide additional insight due to the accuracy and high frequency sampling of these devices.
